# A FELASA Working Group Survey on Fish Species Used for Research, Methods of Euthanasia, Health Monitoring, and Biosecurity in Europe, North America, and Oceania

**DOI:** 10.3390/biology11091259

**Published:** 2022-08-24

**Authors:** Jean-Philippe Mocho, Kristine von Krogh

**Affiliations:** 1 Joint Production System Ltd., Potters Bar EN6 3DD, UK; 2 von Krogh Consult, 1266 Oslo, Norway

**Keywords:** survey, fish research, fish facilities, zebrafish, euthanasia, fish welfare, health monitoring, quarantine, biosecurity, *Danio rerio*

## Abstract

**Simple Summary:**

An international survey was conducted regarding species used for research, methods of euthanasia, health monitoring, and biosecurity in fish laboratories. A total of 145 facilities from 23 countries contributed. Over 80 different species were reported to be used for research, of which zebrafish (*Danio rerio*) was the most common by far. Anesthetic overdose was the preferred method for euthanasia for adults, fry, and larvae not capable of independent feeding. For all developmental stages, the most popular anesthetic compound was tricaine. Around half of the respondents did not perform a completion method to ensure death. One-quarter of the responding facilities did not have a health monitoring system in place. Only a small fraction reported quarantine routines to ensure reliable biological barriers. There was little consensus amongst facilities in how to perform biosecurity measures.

**Abstract:**

An international survey was conducted regarding species used for research, methods of euthanasia, health monitoring, and biosecurity in fish laboratories. A total of 145 facilities from 23 countries contributed. Collectively, over 80 different species (or groups of species) were reported to be used for research, of which zebrafish (*Danio rerio*) was the most common by far. About half of the participating laboratories used multiple species. Anesthetic overdose was the preferred method for euthanasia for adult, fry (capable of independent feeding), and larval (not capable of independent feeding) fish. For all developmental stages, the most popular anesthetic compound was tricaine (MS-222), a substance associated with distress and aversion in several species. Moreover, around half of the respondents did not perform a completion method to ensure death. One-quarter of the responding facilities did not have a health monitoring system in place. While most respondents had some form of quarantine process for imported fish, only a small fraction reported quarantine routines that ensure reliable biological barriers. Furthermore, less than one in five screened fish for pathogens while in quarantine. In sum, there was little consensus amongst facilities in how to perform biosecurity measures. Regarding euthanasia, health monitoring, and biosecurity processes, there is a need for updated and universal guidelines and for many laboratories to adjust their practices.

## 1. Introduction

With the emergence of new scientific tools for imaging and genetic modifications, as well as new interest in environmental matters and toxicology, fish have become major research models in the 21st century. Historically, husbandry and care of aquatic animals in the laboratory have very much been inspired by experience from the aquaculture, pet trade, and public aquaria industries. This has left key questions specific to the needs and ethics of science unanswered, including which methods of euthanasia to apply to allow for humane, fast, and scientifically valid *post-mortem* sampling, as well as the impact of health status on data reproducibility. To address these challenges, FELASA, the Federation of European Laboratory Animal Science Associations, set up two working groups on laboratory fish. One group focused on the humane killing of laboratory fish (excluding the slaughter of fish used for human consumption); the other group focused on the health monitoring of fish in research, in association with AALAS, The American Association for Laboratory Animal Science. To gather knowledge from the research community, the two working groups developed a survey to be distributed to fish laboratories worldwide. The survey had several objectives. The first aim was to make a census of fish species used for research. The second was to assess methods of euthanasia for different life stages of the fish, including completion methods and carcass disposal. The third was to investigate health monitoring practices in fish facilities and identify which pathogens and conditions are considered most important. Finally, the fourth aim was to get insight into biosecurity procedures present in the laboratories. Overall, the survey aimed to help the working groups assess current practices, identify gaps in knowledge, and orienteer the focus of future recommendations.

## 2. Materials and Methods

### 2.1. Survey Design

The survey consisted of an anonymous Google form (Google LLC, Mountain View, CA, USA) in five parts (affiliation of the facility, fish species used, methods of euthanasia, health monitoring, and biosecurity) that were designed predominantly as multiple-choice questions, allowing for several answers (for survey questions, see Supplementary). The remainder of the survey was open-ended questions. Most multiple-choice questions included an “Other” option, which allowed respondents to supply additional info where the given alternatives did not suffice. All questions, except one (regarding which fish species are used in the laboratory), were optional. The total number of questions was 32 and time to fill in the questionnaire was expected to take less than 15 min. The survey link was broadcasted through several networks (i.e., mailing lists and association members) for 6 weeks until 31 May 2018.

### 2.2. Analysis and Presentation of Responses

Although the survey was anonymous, most respondents voluntarily identified their country, email, affiliation, or species of use so that we could cross-check and confirm that every response represented a unique laboratory. The total number of respondents was 145, thus representing 145 fish facilities. Not all participants replied to all questions, and replies per question ranged from 20 to 145 respondents. The number of contributors per question is indicated in the text and/or figures in the results chapter.

As the participants could select more than one alternative for some of the questions, the collective number of answers exceeds the number of respondents in many cases. Support for one alternative was calculated as percentage of the number of respondents to the question, and not the total number of entries, meaning that the total percentage of answers may exceed 100%. When responses to the “Other” options included adequate answers, the information was included in the analysis and is presented in figures, tables, and/or text in the result chapter. No additional statistical analyses were performed on the data. All calculations and graphs were made using Microsoft^®^ Excel^®^ (2013, Microsoft Corporation, Redmond, WA, USA). For each calculation, the resulting percentage was rounded up to the nearest whole number.

## 3. Results

### 3.1. Geographical Distribution of Respondents

**Figure 1:**
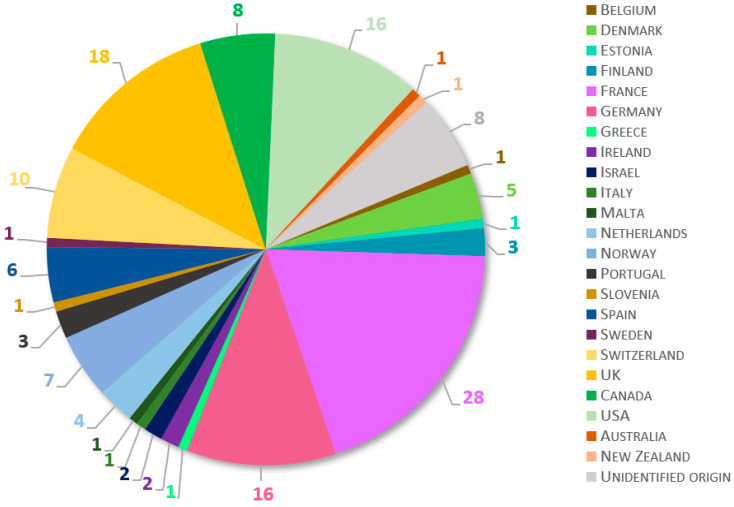
Geographical distribution of laboratories participating in the survey. Total number of contributing laboratories was 145. Eight participants did not declare the location of their facility (“Unidentified origin”).

A total of 145 fish laboratories from at least 23 countries participated in the survey (Figure 1). European laboratories were highly represented, with 111 participants (77%) from 19 countries. French, British, and German establishments provided most of the European responses, with more than 15 contributions each. North American facilities were represented by 24 participants (17%), while two laboratories contributed from Oceania, one from Australia, and one from New Zealand. Because the questionnaire was anonymous, respondents had the option not to indicate their location. In consequence, eight answers were of unidentified origin.

### 3.2. Fish Species Used for Research

**Figure 2:**
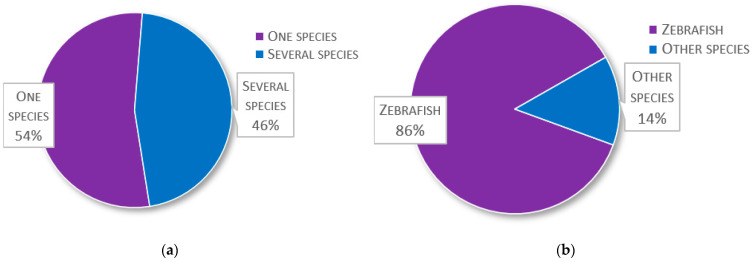

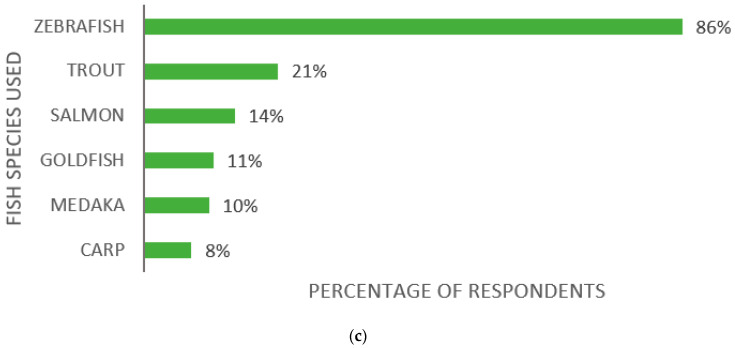
Species composition in survey fish laboratories: (**a**) Proportion of facilities conducting research on either one or two or more species (*n* = 145); (**b**) Proportion of facilities conducting zebrafish (*Danio rerio*) research against facilities using only other species (*n* = 145); (**c**) The most commonly used species amongst the 145 respondents and the percentage of facilities that use them.

The first question considered which fish species the participants used for research, including species used in laboratories and in the field. In the 145 replies, a mixture of scientific and common names, e.g., trout, salmon, or cod, were given (see Table 1). As common names in many cases can refer to several species, the species information is included in the table as they were provided by the respondents. Collectively, 81 species and/or groups of species were reported. Approximately half of the facilities, 54%, conduct research on a single species, whereas 46% conduct research on two or more species (Figure 2a). Among the respondents, the most common research fish is zebrafish (*Danio rerio*), with 125 laboratories (86%) reporting its use (Figure 2b). Other common species used are trout (21%), salmon (14%), goldfish (*Carassius auratus*) (11%), medaka (*Oryzias latipes*) (10%), and carp (8%); other species are used by less than 5% of the laboratories (Figure 2c).

### 3.3. Euthanasia of Research Fish

**Figure 3:**
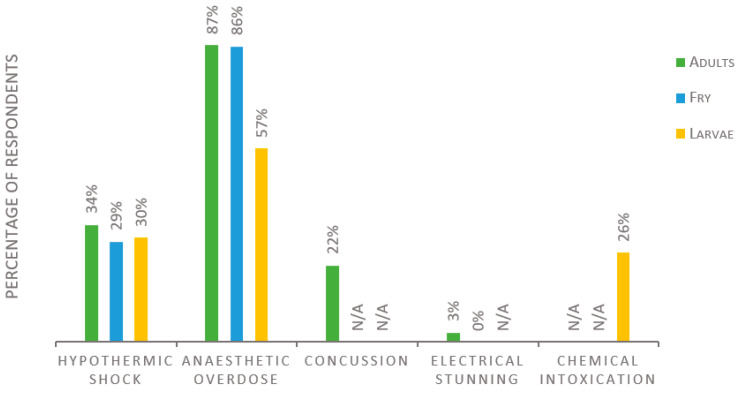
Methods for killing/euthanasia used in fish laboratories according to the developmental stage of the fish, presented as percentage of respondents. Number of respondents was 143, 140, and 138 for adults, fry, and larvae, respectively. Each respondent was allowed to enter multiple answers. N/A; this alternative was not asked for in the survey.

Regarding methods for euthanasia, the survey discriminated between different developmental stages of the fish within three groups: adults, fry, and larvae. The term “fry” is here defined as juvenile fish capable of independent feeding, whereas “larvae” refers to juveniles not yet capable of independent feeding. Preferred methods for killing/euthanasia are summarized in Figure 3. For killing adult fish, 143 replies were received, of which 87% reported using anesthetic overdose, 34% hypothermic shock, 22% concussion/percussive blow to the head, and 3% electrical stunning. Those who use hypothermic shock are all zebrafish or medaka users, and 94% of those who use concussion use species other than zebrafish or medaka. Based on 140 replies for the euthanasia of fry, 86% use anesthetic overdose, and 29% use hypothermic shock, while none use electrical stunning. The questions on larval euthanasia received 138 replies, of which 57% reported using anesthetic overdose, 30% using hypothermic shock, and 26% using chemical intoxication (e.g., bleach, ethanol, hydrogen peroxide). No participants opted to enter any other method for killing/euthanasia than the alternatives listed in the survey.

**Figure 4:**
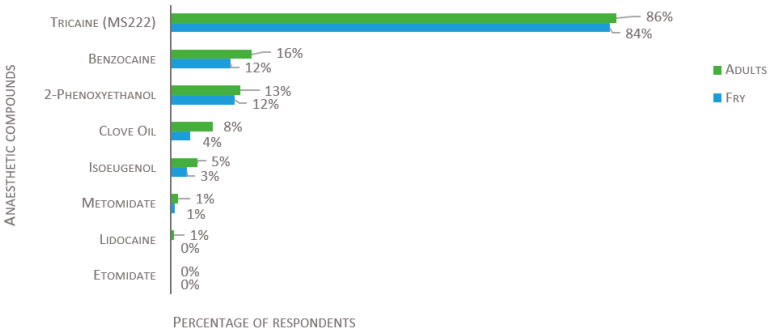
Compounds used to induce an overdose of anesthesia in adults and fry, presented as percentage of respondents (*n* = 134 for adults and 129 for fry). Each respondent was allowed to select multiple answers.

**Figure 5:**
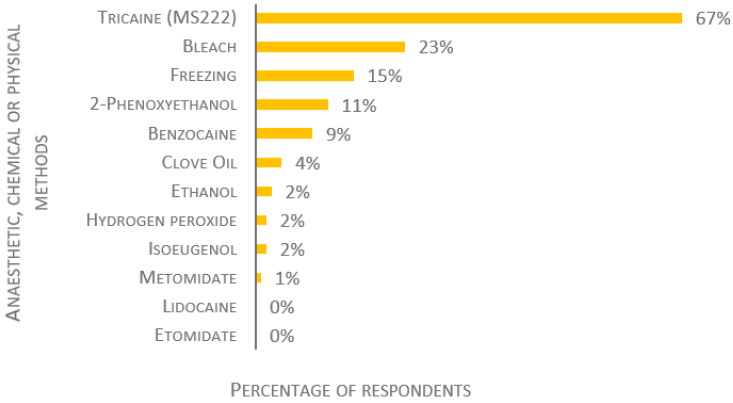
Anesthetic, chemical, and physical methods used to kill larvae, presented as percentage of respondents (*n* = 124). Each respondent was allowed to select multiple answers.

A wide range of compounds were reported for anesthetic overdose induction (Figure 4 and Figure 5). The most popular anesthetic is tricaine (also known as MS-222, TMS, Metacaine, Finquel), used by 86% of the laboratories for adult fish, 84% for fry, and 67% for larvae (of 134, 129, and 124 respondents, respectively). The second most used anesthetics are benzocaine and 2-phenoxyethanol (2-PE); clove oil, isoeugenol, and metomidate are used to a lesser degree. Lidocaine is used by 1% for adult fish, but not for the killing of fry or larvae. Etomidate is not used by any participants, regardless of the developmental stage of the fish. For the killing of larvae, other chemical compounds, such as bleach, ethanol, or hydrogen peroxide, are used by 27%, while freezing is used by 15% (Figure 5).

A method of completion following the killing of adult fish is used by a small majority of respondents (58% of 137). Of those, the most common method is decapitation (52%), followed by destruction of the brain (37%), severing a major blood vessel (15%), or maceration (11%). In addition to the given alternatives, 20% of the respondents reported using freezing as a completion method. For fry, most participants either did not disclose if they used a completion method (61% of 145 participants) or disclosed that they did not use one (10%). Of the 43 participants declaring the use of a completion method for the killing of fry, 44% reported using physical destruction of the brain and/or body through decapitation or maceration, 35% use freezing, 30% use chemical destruction, and 16% use hypothermia.

The last inquiry of this section was about the use of chemical or physical destruction methods of fry carcasses prior to disposal. Of 116 replies, two-thirds freeze fry carcasses before disposal, while one-third destroy the carcasses chemically, using bleach (27 %), prolonged 2-phenoxyethanol immersion (3%), hydrogen peroxide (2%), or ethanol (1%). In addition to the given alternatives, 3% of the respondents reported using autoclaving as a destruction method.

### 3.4. Health Monitoring of Fish

**Figure 6:**
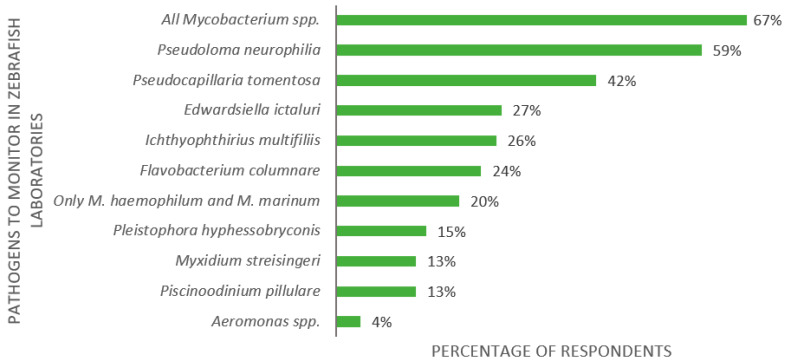
Replies to the multiple-choice question: “What do you think are the main pathogens to monitor in the zebrafish laboratory?”, presented as percentage of respondents (*n* = 123). Each respondent was allowed to select multiple answers.

The third part of the survey was dedicated to health monitoring. The first question regarded pathogens that should be monitored in zebrafish laboratories. The 123 respondents indicated *Mycobacterium* spp. (67%), *Pseudoloma neurophilia* (59%), and *Pseudocapillaria tomentosa* (42%) as the most important zebrafish pathogens (Figure 6). In addition to the given alternatives, five participants (4%) listed *Aeromonas* spp. as significant.

**Figure 7:**
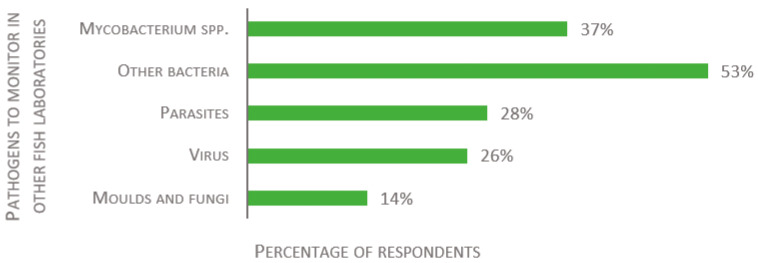
Replies to the open-ended question: “Please list what you consider the main pathogens to monitor in the fish (other than zebrafish) laboratory”, presented as percentage of respondents (*n* = 43).

For fish laboratories housing species other than zebrafish, the respondents were asked to list all important pathogens to monitor. Again, *Mycobacterium* spp. are the most often reported agents (37% of 43 respondents, see Figure 7). The remaining answers covered a wide range of infectious microorganisms, many being host specific. Answers were categorized to build Figure 7. Other oft-mentioned bacteria included *Aeromonas* spp., *Flavobacterium* spp., *Vibrio* spp., and *Pseudomonas* spp. Parasites included, for instance, *Ichthyophthirius multifiliis, Neoparamoeba perurans*, *Pseudocapillaria* spp., and sea lice. Pathogenic microsporidia such as *Pseudoloma neurophilia*, *Glugea* spp., and *Loma* spp., though classified scientifically as fungi, were included here as parasites. Viral pathogens included, for example, carp edema virus (CEV), koi herpesvirus (KHV), infectious hematopoietic necrosis (IHN), and infectious pancreatic necrosis (IPN). Few respondents mentioned fungi and molds, and they were generally not listed by species or groups.

**Figure 8:**
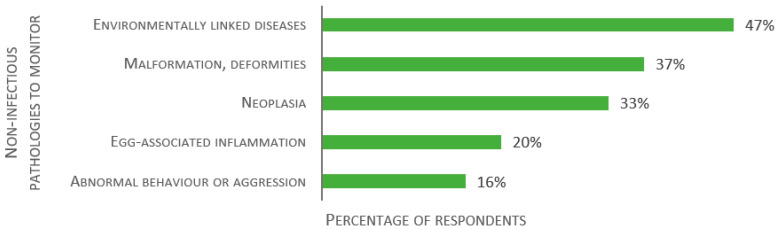
Replies to the open-ended question: “Please list what you consider the main non-infectious diseases to monitor in the laboratory fish”, presented as percentage of respondents (*n* = 49).

Similarly, participants were asked about non-infectious diseases to monitor in laboratory fish (Figure 8). Of 49 valid responses, 47% of the respondents reported environmentally linked diseases (i.e., due to nutrition or water pollution from gas or chemicals), 37% malformations and deformities, 33% neoplasia, 20% egg-associated inflammation, and 16% abnormal behavior or aggression.

A total of 99% of 138 respondents monitor fish mortality, with various systems in place. The only apparent trend is that less than 20% have a procedure for analyzing the mortality data according to the line/strain, location (e.g., holding system), or other specifically defined criteria. About half of the respondents use paper records only, while the other half have databases for mortality records. Mortality is monitored at different developmental stages in different laboratories: 19% monitor mortality all the way from egg harvest selection, 33% from the fry stage, 26% from whenever the fish are received in the laboratory, and 25% monitor only juveniles and adults.

**Figure 9:**
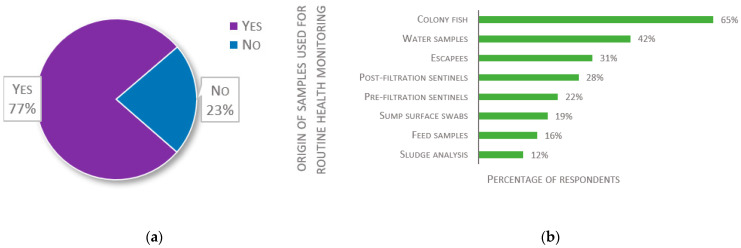
Responses about local health monitoring procedures: (**a**) Responses to the question “Is there any health monitoring system in place in your fish laboratory?” (*n* = 137); (**b**) Responses to the multiple-choice question: “Please select the type or origin of samples you use for routine health monitoring”, presented as percentage of respondents (*n* = 106). Each respondent was allowed to select multiple answers.

Health monitoring systems were declared to be in place by 77% of 137 respondents (see Figure 9a). Of those that have such a system, there is a large variation in the type of samples used for monitoring (see Figure 9b). Most, however, sample colony fish (65%). Other types of fish are also commonly assessed: pre-filtration sentinels, post-filtration sentinels, and escapees are sampled by 22%, 28%, and 31% of screening laboratories, respectively.

The selection of fish sampled for routine health monitoring shows a bias towards older fish, with 43% of screening facilities sampling fertile adults and 30% old fish, whereas only 22% sample juveniles (of 130 respondents). However, 44% use a random selection and 6% sample fish of unknown age. Only a few laboratories (15% of 118) sample fish based on the sex of the animals. A question about how often fish are submitted for routine health monitoring showed that 35% of screening laboratories sample fish once a year or less, 24% every 6 months, 13% every 3–4 months, and 5% monitor more often than quarterly (of 130 responses). Moreover, 40% adapt the submission frequency to clinical signs and mortality of the fish. The number of fish sampled per laboratory averages to 0.7% of the fish population per year (average of 94 responses; range 0.04% to 5.87%).

Environmental samples are also taken by a significant number of screening facilities: 42% sample water, 19% swab sump surface, 12% analyze sludge, and 16% sample feed (see Figure 9b). As to how often environmental or feed samples are taken, 23% reported sampling once a year or less, 23% every 3–6 months, and 21% more often than quarterly. For 48% of these 128 respondents, the frequency depends on clinical signs and mortality, while 38% declared never sampling feed or environment.

In response to which diagnostic assays are used for routine health monitoring, PCR is the favored option (69% of 114 respondents), followed by histopathology (63%). Other techniques such as bacteria culture, necropsy, wet mounts, and serology are used by 39%, 36%, 31%, and 10% of participants, respectively.

**Figure 10:**
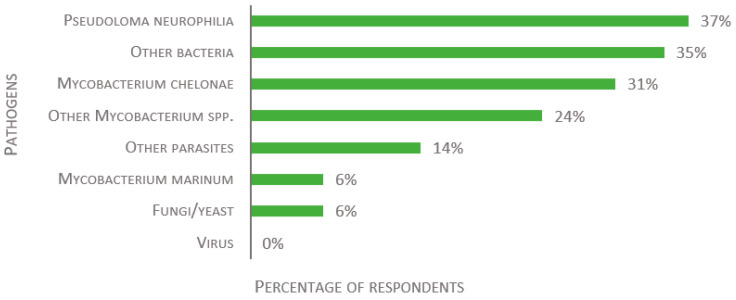
Replies to the open-ended question: “If you are allowed to share the information: which pathogens are present in your fish facility?”, presented as percentage of respondents (*n* = 49).

Finally, laboratories were asked to share their health status data, if possible. Of the 49 received responses, 37% reported that *Pseudoloma neurophilia* was present in their facility, while 14% reported other parasites (Figure 10). Note that, for the sake of this study, pathogenic microsporidia species are listed as parasites and not fungi. *Mycobacterium chelonae* is the most frequent bacteria and was reported by 31% of the respondents. *Mycobacterium marinum* is present in 6% of reporting facilities, while other *Mycobacterium* spp. are present in 24%, and other bacteria in 35%. Fungi are present in 6% of the reporting facilities, while none detected any virus.

### 3.5. Biosecurity in the Fish Laboratory

**Figure 11:**
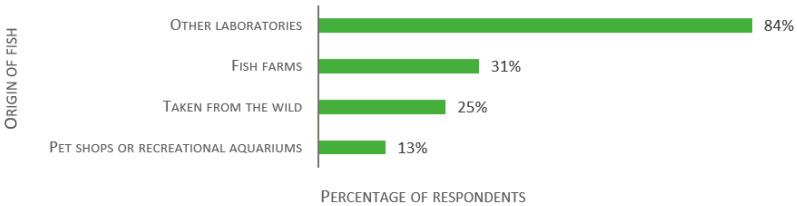
Responses to the multiple-choice question: “Please select the origin of the fish used in your research establishment.”, presented as percentage of respondents (*n* = 138). Each respondent was allowed to select multiple answers.

The last section of the survey focused on biosecurity, starting with import processes and quarantine. Out of 138 respondents, 84% of establishments import fish from other laboratories, 31% from aquaculture, 25% use wild caught fish, and 13% transfer animals from pet shops or recreational aquariums (see Figure 11). In addition, 11% use fish in the wild. Several answers were ticked by 39% of the respondents.

Most laboratories declared having a quarantine process in their facility, while 17% are not equipped with any quarantine (137 respondents). Among the ones benefiting from a quarantine, 64% accept all and any fish in quarantine, 11% allow some fish to skip quarantine, and 15% declared that some fish were not accepted in their facilities regardless of quarantine (114 respondents). Wild caught and pet shop fish are received in quarantine first by 8% and 6% of respondents, respectively. When considering eggs and sperm imports, 38% rear all resulting fish in quarantine, 11% allow some to skip quarantine, and 51% allow some fish from surface-sanitized eggs to skip quarantine (61 respondents).

The quarantine set-ups vary from just a dedicated system in the same room as non-quarantined animals (40%) to a separate, dedicated room (59%) (110 responses). Only a minority indicated having biosecurity measures for their quarantine: restriction on staff movement, e.g., clean to dirty flow (42%), dedicated personal protective equipment (PPE; 33%), flow through system (18%), autoclaving tanks before machine washing (9%), or double bagging exiting items and barrier(s) in place to prevent cross-contamination (6%). Wastewater treatment to prevent environmental contamination equip 24% of quarantines. Of all participants (145), only 27 laboratories (19%) declared screening imported fish while in quarantine. 

**Figure 12:**
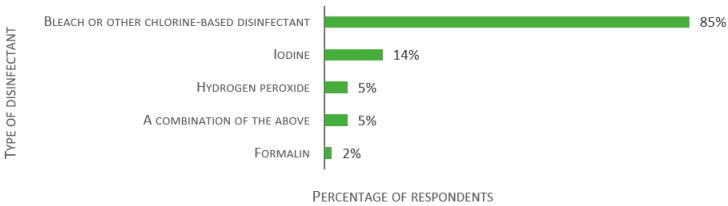
Responses to the multiple-choice question: “Which disinfectant do you use for egg surface sanitation?”, presented as percentage of respondents (*n* = 111). Each respondent was allowed to select multiple answers.

After quarantine, 32% reported transferring any fish or egg to the main holding systems after assessing their health status (of 117 respondents). For 28% of the laboratories, imported fish never leave quarantine. The majority (63%) stated that only surface-sanitized eggs were allowed to enter the main holding systems. Regarding the type of disinfectants that the facilities use for egg surface sanitation, 85% of facilities use bleach or other chlorine-based disinfectant and 14% use iodine (of 111 replies, see Figure 12). Though not an original alternative, formalin was reported by 2%. Sixteen laboratories declared that they did not sanitize egg surfaces.

**Figure 13:**
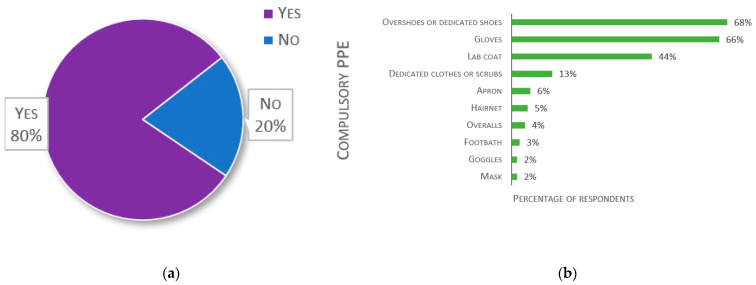
Responses about Personal Protective Equipment (PPE): (**a**) Percentage of respondents having compulsory PPE when entering the aquatic facility (*n* = 137); (**b**) Responses to the multiple-choice question: “Which PPE is compulsory when entering your aquatic facility?”, presented as percentage of respondents (*n* = 117). Each respondent was allowed to select multiple answers.

The second to last question concerned PPE, which are compulsory when entering aquatic facilities. The answers are detailed in Figure 13. Out of 137 responses, 20% of laboratories do not use any PPE (Figure 13a). Of those that have compulsory PPE (*n* = 117), overshoes, shoe-covers, or dedicated shoes are used by 68%. Gloves are required by 66%. Accounting for multiple answers, specific clothing (e.g., apron, lab coat, overalls or dedicated clothes, or scrubs) was declared compulsory by 56% of respondents.

**Figure 14:**
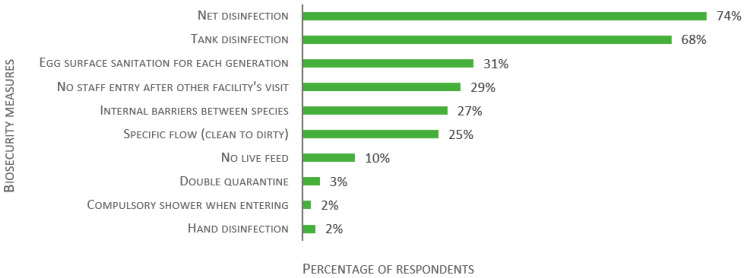
Responses to the multiple-choice question: “Is there any other biosecurity measures or rules in place in your aquatic facility?”, presented as percentage of respondents (*n* = 124). Each respondent was allowed to select multiple answers.

Finally, the survey inquired about other biosecurity measures or rules in place. The responses are detailed in Figure 14 (124 respondents). The majority have disinfection process for nets (74%) and tanks (68%). Other noticeable measures are egg surface sanitation for each generation (31%), disallowing staff entry for a fixed period following visits to another animal facility (29%), specific flow (clean to dirty) between non-quarantine rooms (25%), and internal barriers between species (27%). Though not an original alternative, hand sanitizing was reported by three facilities (2%).

## 4. Discussion

Despite the growing use of fish in research, there is a lack of consensus regarding optimal methods of euthanasia, health monitoring, and biosecurity practices amongst fish facilities. FELASA has appointed two working groups to address these issues and formulate general recommendations. To investigate the current status, the working groups conducted an international survey assessing the subject, to which 145 replies from individual fish research establishments were received.

### 4.1. Limitations of the Study

The survey received a high number of participants, and though the volume of participation likely reflects an interest for such topics by laboratories and suggests that the answers will reflect contemporary procedures, there was an overrepresentation of European facilities amongst the respondents. We therefore assume that the survey broadcast was affected by network limitations, and that the results are biased toward European practices.

Overall, the survey aimed wide and, with one exception regarding zebrafish pathogens, did not include species-specific questions. Given the vast array of species reported to be used for research by the participants, the results can, in most cases not be linked to individual species and must be generally interpreted. However, zebrafish was present in almost nine out of ten facilities, so a bias towards this species is likely present.

The questionnaire did not allow for identification of the position, i.e., researcher, technician, facility manager, etc., of the respondents, which means that the intimate knowledge level of the individual processes that take place in the fish laboratory may vary amongst the participants. We also did not explore the research topics (e.g., cell biology, developmental biology, behavioral biology) that the facilities investigate. Especially in relation to health monitoring, the research topic may be of importance, which will be discussed further in Section 4.4. 

Most of the survey questions were optional to answer, and, as such, some questions were left unanswered by several respondents. There may be many reasons for this: for instance, it might be that a particular question was not relevant for their laboratory, that they did not know the answer, or simply that they chose not to reply. This makes the actual fraction of laboratories having a certain practice hard to conclude. Furthermore, in some cases, the replies were uninterpretable, inconsistent, or by other means unusable. It might be that some survey questions were ambiguously formulated, leading to misunderstandings.

As the participants could check several alternatives, the collective percent of answers exceed 100% per question. This allowed a more exhaustive collection of answers but limited the interpretations for some questions where answers could overlap.

### 4.2. Fish Species Used for Research

The wide geographic range of participants is reflected in the wide range of species that the responding fish laboratories study. Fish used for research include both fresh water and marine species, as well as both wild and domesticated species. Most of the listed species were teleost, but polypteriforms (Senegal bichir), acipenseriforms (sturgeons, paddlefish), and elasmobranchs (sharks, rays, and skates) were also represented. This considerable variation in size, anatomy, and physiology between research fish species will likely require differential practices regarding import processes, housing methods, euthanasia protocols, pathogen monitoring, and biosecurity measures.

The most popular research fish reported was by far the zebrafish, with 86% of the respondents using this species. This finding was not surprising because it is estimated that there are several thousands of zebrafish facilities worldwide [1]. Close to half of the facilities reported using more than one species. For most, but not all, zebrafish was one of those species. Using several species may increase the biosecurity demands on a facility, as pathogens can cause transspecies infections, see Section 4.4 for more details on this topic.

### 4.3. Research Fish Euthanasia

For euthanasia of adults, fry, and larvae, an anesthetic overdose was the overall most popular method amongst the survey participants. Anesthetic overdose can be given both as an immersion or injection, but a distinction between the two methods was not requested in the questionnaire. Nevertheless, the types of anesthetic compounds reported are normally prepared as immersions [2,3]. The high use of anesthesia overdose compared to other killing methods, such as concussion or electrical stunning, might be due to the high number of respondents that use zebrafish. Small laboratory fish are easier to euthanize with an overdose immersion than larger fish or fish used in a field setting.

In general, the responses on euthanasia practices highlight a few topics requiring further investigations. For instance, tricaine is the most used anesthetic, and though the survey was not designed in a manner that allows us to discriminate between laboratories using tricaine as the only method for euthanasia and those that use it as a sedative/anesthetic prior to other methods, such as concussion or decapitation, it does allow us to conclude that, since zebrafish is the most used species, many use tricaine in the euthanasia process of zebrafish. The latter statement is also in concert with previous findings by Lidster et al. (2017) [4]. Tricaine has been described as aversive in zebrafish [5,6,7,8,9], and also as causing avoidance and stress in other species, such as medaka, Atlantic cod (*Gadus morhua*), and Atlantic salmon (*Salma salar*) [10,11]. Is the choice of tricaine then the most humane option to induce an overdose? If not, would other compounds or techniques be more advisable for some species [12]? Based on the results of this survey, better practice for zebrafish euthanasia at all developmental stages have been assessed in two studies, which we refer the reader to for a more exhausting discussion regarding this species [13,14]. It is noteworthy that these experimental data suggest that buffered lidocaine is the best option for zebrafish overdose, whereas only 1% of respondents declared using lidocaine for that purpose in the survey. This may be due to the survey being anterior to these publications. Another explanation is the restrictions in some countries regarding access to (non-)licensed products for veterinary use. However, such restrictions can usually be lifted for the purpose of experimental procedures.

In addition to anesthetic overdose, the Directive 2010/63/EU on the protection of animals used for scientific purposes [15] allows electrical stunning and concussion/percussive blow to the head for research fish euthanasia, while the guidelines from the American Veterinary Medical Association [2] and the Canadian Council on Animal Care (CCAC) [16] also include hypothermic shock as an alternative for some species. Electrical stunning can be used to immobilize fish and is frequently used prior to exsanguination for the slaughter of farmed species [17]. Even though electrofishing is a common method for collecting specimens in the field [18,19], electrical stunning currently has few applications in the laboratory, and the survey answers indicate that such practice is sporadic amongst the respondents: 3% for adults and 0% for fry. An advantage of electrical stunning is that it can be suitable for both small and large fish and fitting for both individuals and groups, although it demands special equipment and trained operators. Most studies on the efficacy and humanity of the electrical stunning procedure are aimed towards farmed, not research, fish [12,20,21,22]. However, a recent study using specially designed equipment intended for the laboratory setting indicates that electrical stunning can be a very effective and stress-free method for the euthanasia of zebrafish larvae [13]. Should fish experience during electrical stunning be explored further? Can more equipment be developed to better suit laboratory conditions?

For adult fish, concussion or percussive blow to the head was used by 22% of the participants. Such procedures are usually performed on large and docile or sedated fish on an individual basis. In a farm setting, automatic stunning devices are available [23]; in the laboratory, a manual approach using a priest is the common standard. Percussion requires high skills and intensive training of the operators but can be a time efficient and humane method if performed correctly. It can be fitting for scientific procedures needing tissue or cells unaffected by anesthetics; however, it could be detrimental to, for instance, brain tissue. Furthermore, this method is not suitable for all fish—e.g., it would be challenging to perform precisely on small fish, fry, or larvae—and it is not suitable for terminating a group of animals at once. Conversely, hypothermic shock is an option when killing groups of smaller, especially tropical, fish. It is an easy to operate, chemical-free, practical, fast, and inexpensive method [8,24,25,26]. Still, fish experience during the ice-chilled water immersion is unknown and signs of aversion are not rare [8,13,26]. Furthermore, this method is not useful for large and, in particular, cold-water adapted fish, as it may take an unacceptably long time to induce loss of consciousness and may induce aversive behavior [2,21,27,28]. About one-third of the survey respondents indicated using hypothermic shock for adults, fry, and larvae. All participants using hypothermic shock also reported using medaka and/or zebrafish as research species, so it is likely that this method is most often performed using small tropical fishes. Hypothermic shock is not listed in the current EU directive but is approved by the AVMA guidelines for small tropical fish [2]. AVMA also recommend this method for embryos and larvae, but, here, there is room for improvement in the guidelines, as previous studies indicate that the efficacy of hypothermic shock can be low or insufficient for the euthanasia of zebrafish embryos, larvae, and fry before 16 days post fertilization [13,26,29]. There is no published data assessing an age-dependent efficacy for each fish species, e.g., other small tropical fish such as medaka. Therefore, we do not know from which stage of development hypothermic shock can be efficacious in other fish species or how long the exposure to chilled water should be to ensure death and prevent recovery. Bearing in mind that each species would undergo different developmental time points, data from zebrafish fry are not translatable to other species.

Larvae not capable of independent feeding are not protected by current legislation, hence there are less detailed guidelines for euthanasia for this developmental stage. While the EU directive does not distinguish between different protected developmental stages or fish species, the AVMA guidelines are more nuanced and proposes some different options based on age and species. For larval euthanasia, most survey participants use an anesthetic overdose, as described above, while over one-quarter use chemical intoxication, both being approved methods by AVMA. Moreover, 15% of the participants reported using freezing as a method for larval euthanasia. This is not deemed an acceptable method for euthanasia neither by the EU directive nor AVMA. However, the design of the questionnaire did not allow a distinction between freezing used as an initial or an adjunctive method, the latter being an approved method by AVMA [2]. We strongly recommend to induce an overdose of anesthesia before freezing and to avoid chemical intoxication on larvae reaching sentience. Ideally, a scientifically validated species- and life stage-specific non-aversive anesthetic protocol would be used [13].

According to both the EU directive and AVMA, the killing of animals shall be completed by confirmation of permanent cessation of the circulation or destruction of the brain. The questionnaire suggests that almost half (42%) of the laboratories do not follow these guidelines for adult fish. The numbers for fry were somewhat difficult to interpret but are likely even higher. Without a secondary procedure, it is possible that stunned or anesthetized animals may regain consciousness [5,13,20,26]. It is therefore advisable for these laboratories to review their practices because confirming death and ensuring a humane euthanasia process is of great importance to animal welfare. 

The result from the survey echoes the previous statement in that there is a gap of knowledge and consensus regarding fish euthanasia processes, and the topic would probably benefit from being discussed further within the fish user community. For instance, what would be the anesthetic of choice to euthanize fish of all developmental stages and species? How can we humanely kill fry and larvae? Which completion process would be advisable to comply with current legislations? These questions have started to be addressed [12,13,14], and it is the objective of the FELASA working group to address them further.

### 4.4. Health and Biosecurity in the Laboratory

Good health is an essential, but not unique, component of good animal welfare. To maintain the fish colonies in good health and warrant reproductive success and unambiguous experimental data, sound husbandry standards and emphasis on stress reduction must be in place [30,31], as well as biosecurity measures to limit pathogen exposure. Important arenas for biosecurity precaution are health monitoring, import processes, mixing of species, and protection of operators.

A thorough health monitoring system consists of daily observation of the fish, registration of mortality, and screening for clinical signs and pathogens [15,16,32,33,34]. Almost all survey participants measure mortality in the fish population, giving them the ability to detect unusual mortality patterns that could indicate pathogen outbreaks, uncontrolled husbandry parameters, or other health issues (e.g., harmful phenotype). However, the survey revealed that there is no common way to register mortality amongst facilities. Less than one-fifth reported having procedures for analyzing mortality data according to, for instance, line/strain or holding systems, which would be useful to reveal signs of inbreeding, locate the source of the problem, etc. Further, less than 20% include eggs and larvae in their mortality records, and only one-third include fry. Theoretically, high mortality in the younger fish population could indicate problems in the laboratory that could affect future health for their surviving relatives. While recording mortality at all stages may provide useful information, it does not suffice as the only health monitoring measure. It is vital to screen the colonies for clinical signs to discover sick or injured fish and take appropriate action [35,36]. It should also be stressed that fish not displaying symptoms of disease does not exclude infectious agents from being present [37,38,39]. Both infectious and non-infectious diseases may affect animal welfare and scientific outcome before reaching clinical levels, and well before affecting mortality levels [38,40]. Thus, routine screening for the presence of subclinical levels of pathogens is recommended [34]. Resources on fish diseases and pathogens may be found online at the Zebrafish Information Network (ZFIN; zebrafish.org) or the Office International des Epizooties (World Organisation for Animal Health (OIE); oie.int) [34].

Species from the *Mycobacterium* genus and the microsporidium *Pseudoloma neurophilia* are commonly found in fish facilities [37,38,41,42,43,44] and were also the most frequently detected species amongst the survey facilities. Still, about one-quarter of the participants reported not having pathogen surveillance systems in place. For these facilities, microbes may proliferate undetected and affect both fish well-being and experimental results [38]. Several recent studies give examples of the latter. For instance, in zebrafish, *P. neurophilia* has been shown to affect both molecular and behavioral endpoints [45,46,47,48]. Using the Epitools online software [49], the number of fish needed for screening can be determined based on pathogen prevalence, colony size, and test specificity (see Table 2). Amongst the survey facilities, the 77% that perform routine surveillance reported screening an average of 0.7% of the fish population per year. According to Epitools, assuming perfect test specificity, this number of fish would suffice to detect a pathogen with 5% threshold prevalence in a 10,000 fish colony, but is too low to detect less abundant microbes. That does not mean that facilities should screen more, but rather that it is important to understand the limits of a screening program. For example, to detect a pathogen with 1% prevalence in a population of 10,000, 311 colony fish would be needed (equaling 3.11% of the population). Further, many facilities have smaller fish populations, which demands an even higher fraction of the population to detect low microbe prevalence.

While most of the participants use colony fish for health monitoring, some also include sentinel fish. The use of prefiltration sentinel fish can reduce the number of animals needed for screening because they are exposed to unfiltered effluent water from colony fish tanks and hence are at a maximized risk of being infected [34,50,51]. To further decrease the number of sacrificed fish, biofilms, sump surface swabs, feed, water, or sludge can be used for compensatory sampling [36,51,52]. Using such samples can be an effective and cost saving method to detect the presence of some pathogens. Among the survey participants, 42% include water samples for screening, and other environmental samples are included by less than 20%. This is therefore an area where facilities can improve their monitoring routines without using additional animals, and at a relatively low cost [34].

In combination with a surveillance program for microbiological status in the laboratory, quarantine and screening any new entries into the facilities are important measures to preserve the health status. Transfer of fish between laboratories is common and of great value to research progress. For instance, thousands of medaka and zebrafish transgenic lines are exchanged between collaborating institutions worldwide. According to survey participants, 84% import new fish from other laboratories. Importing fish is associated with significant risk of also importing pathogens. While fish from resource centers are usually intensively screened [44], wild or pet shop fish are likely to have unknown health statuses [41]. Further, as nearly one-quarter of the survey respondents do not perform microbiological inspections, importing fish from any of these laboratories can be hazardous. Quarantine ensures that fish are not moved into or out of facilities without caution. However, 17% of the surveyed facilities do not have any form of quarantine, and for 40% of those with a quarantine in place, it consists only of a separate system in the same room as non-quarantined animals. To prevent cross-contamination between different populations of fish, reliable biosecurity barriers need to be in place; such biosecurity measures are only present in 6% of survey facilities. Furthermore, less than one-fifth screen fish while in quarantine. This, along with the fact that more than half of the respondents accept all fish into quarantine, regardless of information on health status, should be a cause for concern [53].

About half the surveyed facilities work with multiple species, which represents an increased chance of pathogen transmission. For example, many viruses belonging to the Megalocytivirus genus have broad host specificity and can cross species boundaries [54,55]. Similar, many Mycobacterium species have multiple host species [37]. In addition to fish, personnel can be affected by laboratory health status through transmission of pathogens from fish to humans, i.e., zoonotic disease. For instance, it is well documented that *Mycobacterium marinum* can infect humans and cause skin lesions [37,39,52,56]. The use of PPE protects both fish and personnel. Changing PPE between interactions with different species, or when moving from quarantined to non-quarantined animals, can reduce the risk of pathogen transfer. While most survey participants use PPE such as dedicated shoes, gloves, or specific clothing when entering the aquatic facility, one in five reported having no compulsory PPE. More than one-quarter also do not disinfect nets, tanks, etc., a simple biosecurity measure that would be easy to implement [53].

Because of advances in diagnostic technologies, increasing knowledge, and new emerging or discovered pathogens, biosecurity is a dynamic discipline. Standardizing health monitoring processes and how these are reported in scientific papers may aid researchers in keeping up to date and obtaining more reproducible results. Despite monitoring and reporting being called for on several occasions over the last decades [32,34,40,50,53,57,58], some may still face difficulty implementing standard operational protocols. This might be due to staffing challenges, limited knowledge of the potential impact of pathogens on animal health, research results, and personnel, as well as the financial burden of routine screening [40]. While pathogen-free fish may not be desirable in all cases, as pathogens may be present in wild populations and thus represent a truer state of the animal, health status should be reported when publishing scientific results. Even if the effect of a particular pathogen is unknown, thorough reporting will likely reduce the number of animals needed for research and contribute to the 3Rs (Replace, Reduce, Refine; first described by Russel and Burch (1959)) [59]. Is it time for health conditions to be included into research reports in the same manner husbandry conditions are? Should such reporting be made mandatory by research journals? Based on the information that this survey provided on current practices, the FELASA-AALAS working group has prepared two manuscripts with recommendations for health monitoring, management of zoonotic hazards, quarantine, and biosecurity in fish research laboratories [34,53]. The aim of these recommendations is to aid facilities in exchanging fish safely by developing strategic health management and biosecurity protocols, reporting health status and husbandry practice in standardized templates, and strengthening their practices.

## 5. Conclusions

This survey allows international assessment of current practices in fish laboratories regarding euthanasia, health monitoring, and biosecurity, and highlights topics for which improvements and refinements should be explored. For euthanasia, most existing guidelines separate their recommendations poorly on fish species and life stages. Considering the vast number and variety of fish species used in research, there is clear need for more nuanced guidelines. These would be inspired by more research on species- and life stage-specific euthanasia. Considering the potential impact of pathogens on the variability of experimental results, the consensus amongst laboratories on health monitoring is weak, especially considering the high number of facilities not performing any pathogen surveillance, which gives cause for concern. Similarly, quarantine and biosecurity measures have room for improvement. Based on the results of the survey, the FELASA and AALAS working groups can focus on addressing some of the highlighted challenges and propose practical recommendations for good practices.

## Figures and Tables

**Table 1:** List of species and/or groups of fishes used for research by survey participants.

African sharptooth catfish (*Clarias gariepinus*)	Eel	Pike (*Esox lucius*)
American paddlefish (*Polyodon spathula*)	European grayling (*Thymallus thymallus*)	Plaice
Anchovy	Flathead grey mullet (*Mugil cephalus*)	Plainfin midshipman (*Porichthys notatus*)
*Astyanax mexicanus*	Flounder	*Pseudochondrostoma duriense*
Atlantic cod (*Gadus morhua*)	Gilt head sea bream (*Sparus aurata*)	Ray
Ballan wrasse (*Labrus bergylta*)	Goby	Roach
Bigeyes	Golden orfe (*Leuciscus idus*)	Rockling
Bluegill sunfish (*Lepomis macrochirus*)	Goldfish (*Carassius auratus*)	Salmon
Bullhead	Gourami	Sculpin
Butterfish	Gudgeon	Sea bass
Carp	Guppy	Senegal bichir (*Polypterus senegalus*)
Catfish	Hagfish	Shark
Charr	John Dory	Shortfin molly (*Poecilia mexicana*)
Cichlids (other than tilapia)	Killifish	Skate
Clownfish	Knifefish	Smelt
Cobia (*Rachycentron canadum*)	Lamprey	Snapper
Cod	Leather jacket (*Oligoplites saurus*)	Sole
Common barbel (*Barbus barbus*)	Luciobarbus bocagei	Stickleback
Common bream (*Abramis brama*)	Lumpsucker	Sturgeon
Common chub (*Squalius cephalus*)	Marblefish	Sunbleak (*Leucaspius delineatus*)
Common dab (*Limanda limanda*)	Meagre (*Argyrosomus regius*)	Tench (*Tinca tinca*)
Common rudd (*Scardinius erythrophthalmus*)	Medaka (*Oryzias latipes*)	Tilapia
Common sunfish (*Lepomis gibbosus*)	Minnow (other than zebrafish)	Trout
Common triplefin (*Forsterygion lapillum*)	Parore	Tuna
Dragonet	Peacock blenny (*Salaria pavo*)	Turbot
Drummer (*Pogonias cromis*)	Perch	Wolffish
Eastern mudminnow (*Umbra pygmae*)	*Phreatichthys andruzzii*	Zebrafish (*Danio rerio*)

**Table 2:** Screening sample sizes with varying threshold prevalence and population size. Based on a test specificity of 100%, test sensitivity of 95%, and a confidence interval of 95%. Calculations were made at https://epitools.ausvet.com.au/ (accessed on 23 August 2022) [49].

Population Size	Prevalence				
	0.5%	1%	5%	10%	50%
500	333	238	60	31	7
1000	475	273	62	32	7
5000	595	307	63	32	7
10,000	613	311	63	32	7
100,000	629	315	64	32	7
1,000,000	631	316	64	32	7
∞	630	314	62	31	5

## Data Availability

Data are available on request.

## Supplementary Materials

The following supporting information can be downloaded at: https://www.mdpi.com/article/10.3390/biology11091259/s1.
