# Supplementary material for: A FELASA Working Group Survey on Fish Species Used for Research, Methods of Euthanasia, Health Monitoring, and Biosecurity in Europe, North America, and Oceania
Source: Biology (Basel). 2022 Aug 24;11(9):1259. doi: 10.3390/biology11091259 (PMC9495953; doi:10.3390/biology11091259)
Supplement: Supplementary file 1 [file biology-11-01259-s001.zip › biology-1860567-supplementary.pdf]

Survey on Fish used for Research

I. Affiliation, email

II. Fish species used for research:
The main aim of the survey is to establish a census of fish species used for research. Please be exhaustive.

1. In your laboratory, which fish species is used for research (including in the wild)?

III. Laboratory fish euthanasia:
For each question you may tick more than one box.

1. How do you kill ADULT fish in your laboratory?

□ Concussion/percussive blow to the head

□ Electrical stunning

□ Hypothermic shock

□ Anaesthetic overdose

□ Other:

2. Which anaesthetic do you use to induce an overdose of anaesthesia for an ADULT fish?

□ Overdose of anaesthesia is not used in my lab for adult fish

□ 2-Phenoxyethanol

□ Benzocaine

□ Clove Oil

□ Etomidate

□ Isoeugenol

□ Lidocaine

□ Metomidate

□ Tricaine (MS222)

□ Other:

3. Is the killing of ADULT fish followed by one of these completion methods?

□ No completion method

□ Maceration

□ Decapitation

□ Destruction of the brain

□ Severing a major blood vessel e.g. caudal peduncle or gills

□ Other:

4. How do you euthanize FRY CAPABLE of independent feeding?

□ Hypothermic shock

□ Electrical stunning

□ Overdose of anaesthesia

□ Other:

5. Which anaesthetic do you use to induce an overdose of anaesthesia for FRY?

□ Overdose of anaesthesia is not used in my lab for fry

□ 2-Phenoxyethanol

□ Benzocaine

□ Clove Oil

□ Etomidate

□ Isoeugenol

□ Lidocaine

□ Metomidate

□ Tricaine (MS222)

□ Other:

6. If the killing of FRY is followed by a completion method, please detail the used method(s):

7. Please tick any chemical or physical method(s) used to destroy FRY carcasses before disposal:

□ Ethanol

□ Bleach

□ Hydrogen peroxide

□ Prolonged 2-phenoxyethanol immersion

□ Freezing

□ Other:

8. How do you euthanize LARVAE NOT CAPABLE of independent feeding?

□ Chemical intoxication e.g. bleach, ethanol, hydrogen peroxide

□ Hypothermic shock

□ Overdose of anaesthesia

□ Other:

9. Which anaesthetic, chemical or physical method do you use to kill LARVAE?

□ No chemical or anaesthetic used

□ Bleach

□ Ethanol

□ Hydrogen peroxide

□ Freezing

□ 2-Phenoxyethanol

□ Benzocaine

□ Clove Oil

□ Etomidate

□ Isoeugenol

□ Lidocaine

□ Metomidate

□ Tricaine (MS222)

□ Other:

IV. Health monitoring of fish:

For each question you may tick more than one box.

1. What do you think are the MAIN pathogens to monitor in the ZEBRAFISH laboratory?

□ All *Mycobacterium spp*. should be monitored

□ Only *Mycobacterium haemophilum* and *Mycobacterium marinum* should be monitored

□ *Edwardsiella ictaluri*

□ *Pseudocapillaria tomentosa*

□ *Pseudoloma neurophilia*

□ *Flavobacterium columnare*

□ *Myxidium streisinger*

□ *Pleistophora hyphessobryconis*

□ *Piscinoodinium pillulare*

□ *Ichthyophthirius multifilis*

□ Other:

2. Please list what you consider the MAIN pathogens to monitor in the fish (other than zebrafish) laboratory.

3. Please list what you consider the MAIN NON-INFECTIOUS diseases to monitor in the laboratory fish.

4. Is there any health monitoring system in place in your fish laboratory?

□ Yes

□ No

5. How do you monitor mortality in the fish population?

□ Paper record only

□ Database

□ From egg harvest selection

□ From capable of independent feeding age

□ From whenever they are received in the lab

□ To juvenile or adult age

□ To their death or euthanasia

□ Mortality data is available according to the animal type only (e.g. strain dependent)

□ Mortality data is available according to the location only (e.g. per holding system)

□ Mortality data is available according to criteria we define

□ Mortality is not monitored

□ Other:

6. Please select the type or origin of samples you use for routine health monitoring.

□ Pre-filtration sentinels

□ Post-filtration sentinels

□ Escapees

□ Colony fish

□ Water samples

□ Sump surface swabs

□ Sludge analysis

□ Feed samples

□ No sample tested

□ Other:

7. Which diagnostic assays do you use for routine health monitoring?

□ Bacteria culture

□ Histopathology

□ PCR

□ Post-mortem

□ Wet mounts and other fast macro or microscopic analysis

□ Serology

□ Other:

8. Please tick the age of the fish sampled for routine health monitoring.

□ Juveniles

□ Fertile adults

□ Old fish

□ Random selection

□ Selection of specific age groups

□ Unknown

□ No fish are sampled

□ Other:

9. Are fish selected according to their sex?

□ Yes

□ No

10. How often are fish submitted for routine health monitoring?

□ No fish are sampled

□ Once a year or less

□ Every 6 months

□ Every 3-4 months

□ More often than quarterly

□ Depends on clinical signs and mortality

11. How many fish for which population size do you send for each routine submission? For example, the answer could be 10 screened fish for a 2000 fish system quarterly or 60 tested fish for 5000 fish once a year.

12. How often are environmental or feed samples taken?

◻ Environment and feed are never sampled

◻ Once a year or less

◻ Every 6 months

◻ Every 3-4 months

◻ More often than quarterly

◻ Depends on clinical signs and mortality

13. If you are allowed to share the information: which pathogens are present in your fish facility and what are the main diseases that you detect?

V. Biosecurity in the fish lab:

For each question you may tick more than one box.

1. Please select the origin of the fish used in your research establishment.

◻ Other laboratories

◻ Pet shops or recreational aquariums

◻ Fish farms

◻ Taken from the wild

◻ Some fish are used in the wild

2. Which animals enter quarantine?

□ No quarantine

□ All/any fish are accepted in quarantine

□ Some fish are not accepted in the facility at all (not even quarantine)

□ Some fish may be allowed to skip quarantine

□ All wild caught fish are received in quarantine first

□ All pet shop fish are received in quarantine first

□ All fish from imported eggs and sperm are reared in quarantine

□ Some fish from imported eggs and sperm may skip quarantine

□ Some fish from imported eggs and sperm may skip quarantine following surface sanitation

3. What is your quarantine set-up?

□ No quarantine

□ Just a dedicated system in the same room than non-quarantined animals

□ Dedicated room

□ Flow through system

□ Recirculating system

□ Restriction on staff movement e.g. clean to dirty flow

□ Dedicated PPE

□ Tanks are autoclaved then machine washed

□ Exiting items are double bagged and there is a barrier in place to prevent cross-contamination

□ Wastewater is treated to prevent environmental contamination

4. If imported fish are screened for specific pathogens while in quarantine, please describe which pathogens and how.

5. Which fish are transferred from quarantine to the main holding systems?

□ Any fish or egg after the assessment of their health status

□ Imported fish never leave quarantine

□ Only surface sanitized eggs enter the main holding systems

6. Which disinfectant do you use for egg surface sanitation?

□ None

□ Bleach or other chlorine-based disinfectant

□ Iodine

□ Hydrogen peroxide

□ A combination of the above

□ Other:

7. Which Personal Protective Equipment (PPE) is compulsory when entering your aquatic facility?

□ No compulsory PPE

□ Overshoes or dedicated shoes

□ Gloves

□ Apron

□ Lab coat

□ Overalls

□ Use of dedicated clothes or scrubs

□ Hairnet

□ Mask

□ Other:

8. Is there any other biosecurity measures or rules in place in your aquatic facility?

□ Compulsory shower when entering

□ Not having visited another animal facility in the last 12-48 hours or more

□ No live feed

□ Internal barriers between species

□ Specific flow (clean to dirty) between non-quarantine rooms

□ Double quarantine i.e. only surface sanitized 3rd generation eggs leave quarantine

□ Net disinfection

□ Tank disinfection

□ Egg surface sanitation for each generation

□ Other:
